# Safety and efficacy of intravitreal dexamethasone implantation along with phacoemulsification and intraocular lens implantation in children with uveitis

**DOI:** 10.1186/s12348-024-00440-y

**Published:** 2024-10-24

**Authors:** Hui Feng, Weixin Chen, Jianzhu Yang, Haorong Kong, Hongyu Li, Meng Tian, Jing Mo, Yuan He, Hong Wang

**Affiliations:** Beijing Ophthalmology and Visual Sciences Key Laboratory, Beijing Tongren Eye Center, Beijing Institute of Ophthalmology, Beijing Tongren Hospital, Capital Medical University, 1 Dongjiaominxiang Street, Dongcheng District, Beijing, China

**Keywords:** Pediatric uveitis, Cataract surgery, Intravitreal dexamethasone implantation

## Abstract

**Purpose:**

To evaluate the safety and efficacy of intravitreal dexamethasone implantation during phacoemulsification and intraocular lens implantation in pediatric uveitis.

**Methods:**

A retrospective analysis was conducted on pediatric uveitis patients undergoing phacoemulsification and intraocular lens implantation with intravitreal dexamethasone implantation. Patients with a minimum follow-up of 6 months were included. Primary outcome measures included ocular inflammation, intraocular pressure (IOP), best-corrected visual acuity (BCVA), and worsening of uveitis.

**Results:**

36 eyes of 28 patients were ultimately included in this study. The mean preoperative BCVA was 1.00 (0.40–1.50) LogMAR. BCVA significantly improved to 0.40 (0.20–0.54) LogMAR at 1 month postoperatively (*P* = 0.006), further improving to 0.30 (0.20–0.40) LogMAR at 3 months postoperatively (*P* = 0.001). BCVA remained stable at 0.30 (0.20–0.70) LogMAR at 6 months postoperatively (*P* = 0.005). Mean IOP showed no statistically significant difference during the follow-up period of three to six months after surgery. Eight children experienced recurrence of ocular inflammation during the 6-month follow-up period. No cases of worsening macular edema, glaucoma, or elevated IOP were observed in any patient.

**Conclusion:**

Intravitreal dexamethasone implantation during phacoemulsification and intraocular lens implantation is a safe and effective method for preventing and treating postoperative inflammation in children with uveitis.

## Introduction

In developed countries, uveitis accounts for 10-15% of causes of blindness. Although pediatric uveitis is relatively rare, constituting only 5-10% of all uveitis cases, the complications threatening vision are common, leading to a high rate of visual impairment [1–3]. Previous studies have reported a prevalence of cataracts in pediatric uveitis of 44.2%, with up to 69% of pediatric patients developing uveitis-associated cataracts over time [4]. Cataracts and uveitis can profoundly impact the vision of these children. Cataract surgery poses particular challenges in pediatric uveitis because it can exacerbate inflammation and lead to various serious complications. Traditionally, cataract surgery is performed at least 3 months after inflammation has subsided, with favorable outcomes [5, 6]. However, despite preoperative control of inflammation, children may experience various postoperative complications due to inflammation recurrence, such as macular edema, posterior capsule opacification (PCO) causing visual axis blur, and glaucoma.

Cataract surgery is another factor that triggers a cascade of inflammatory reactions; therefore, it is usually recommended to increase the dose of corticosteroids perioperatively [7, 8]. However, escalating drug doses, particularly corticosteroids, can cause systemic side effects, leading to growth retardation, Cushing’s syndrome, behavioral changes, and associated psychosocial issues.

Dexamethasone implant (Ozurdex^®^, Allergan Inc., Irvine, California) is a sustained-release intravitreal implant containing 700 µg of dexamethasone. Intravitreal corticosteroid injection maximizes drug delivery to ocular target tissues and minimizes systemic adverse effects associated with increased oral drug dosages. Jinagal et al. [9]observed the safety and efficacy of intravitreal dexamethasone implantation in six children with juvenile idiopathic arthritis (JIA) -associated uveitis undergoing phacoemulsification and posterior chamber intraocular lens (IOL) implantation. Significant improvements in BCVA were observed at 1, 3, and 6 months postoperatively, with no occurrences of elevated intraocular pressure (IOP), uveitis recurrence, or worsening of cystoid macular edema. However, there remains a lack of large-scale clinical studies observing the safety and efficacy of dexamethasone implantation in pediatric uveitic cataract surgery.

In this retrospective study, we report on the occurrence of complications, treatment modalities, and the safety and efficacy of intravitreal dexamethasone injection during cataract surgery in pediatric uveitis patients seen at Beijing Tongren Hospital for controlling ocular inflammation.

## Methods

### Patients and diagnosis

Researchers reviewed the clinical records of all pediatric uveitis patients seen at Beijing Tongren Hospital’s uveitis clinic from January 2020 to December 2023. Diagnosis of pediatric uveitis was based on the International League of Associations for Rheumatology (ILAR) diagnostic criteria below 16 years of age [10]. The study population also included children with significant cataracts who underwent phacoemulsification surgery and intraocular lens implantation, with intravitreal dexamethasone implantation during surgery. All children underwent a minimum follow-up of 6 months. Preoperative control of inflammation (less than 1 + cells) for at least 3 consecutive months and best corrected visual acuity (BCVA) < 20/50 or worse were prerequisites for surgery. Factors such as risk of amblyopia and preoperative visual acuity were simultaneously considered when determining the timing of surgery. Children who did not receive intraocular lens implantation or had a follow-up period of less than 6 months were excluded from the study. The study was approved by the Institutional Research and Ethics Committee of Beijing Tongren Hospital and followed the principles of the Helsinki Declaration. Written informed consent was obtained from adult patients and their parents or legal guardians before enrollment.

### Clinical examination

All patients underwent comprehensive ophthalmic examinations, including BCVA, slit-lamp biomicroscopic examination, intraocular pressure (IOP) measurement, indirect ophthalmoscopy, fundus fluorescein angiography (FFA), and enhanced depth imaging optical coherence tomography (EDI-OCT) examination. Treatment details, systemic diseases, ocular complications, and follow-up duration were also documented for all patients. Anterior segment inflammation was assessed according to the Standardization of Uveitis Nomenclature (SUN) criteria [11]. Additionally, patients underwent thorough physical examinations and laboratory blood tests (including hepatitis serology, and screening for syphilis and HIV antibodies). Each patient underwent tuberculosis screening through both the Purified Protein Derivative (PPD) and QuantiFERON-TB Gold test (QFT).

### Patient management

Patients underwent ophthalmic examinations on postoperative day 1, at 2 weeks, 1 month, 3 months, and 6 months. Initially, all children received topical ocular therapy, including 1% prednisolone acetate eye drops six times daily, gatifloxacin eye gel one time at bedtime, tobramycin eye drops four times daily, and recombinant bovine basic fibroblast growth factor eye drops four times daily, gradually tapered based on postoperative ocular inflammatory response. At each visit, patients underwent a complete ophthalmic examination, including BCVA, IOP, slit-lamp biomicroscopic examination of the anterior segment, cell and flare assessment, and fundus examination. BCVA was evaluated using a standard logarithmic visual acuity chart and converted to LogMAR format. Visual acuity assessment included BCVA at the initial visit, preoperative visual acuity referring to BCVA at the preoperative clinic visit before cataract surgery, and BCVA at each follow-up visit postoperatively.

### Surgical procedure

All surgeries were performed by the same surgeon (Hong Wang) following strict sterile techniques. Patients received written informed consent before surgery. All surgeries were performed under general anesthesia. Two corneal side ports were created at the 2 o’clock and 10 o’clock positions, and the main incision was made using a 3.0 mm disposable corneal knife. Anterior chamber formation was achieved using high-viscosity viscoelastic (1.7% sodium hyaluronate). In cases where pupillary dilation was inadequate due to posterior synechiae, viscoelastic was injected into adherent iris or posterior synechiae were released using an iris repositor. A 5–5.5 mm capsulorhexis was made using capsulorhexis forceps. Standard steps of pediatric lens aspiration were performed. All patients received foldable hydrophobic acrylic IOL (A1UL22^®^, Eyebright, Beijing, China) implantation. To prevent postoperative iris posterior synechiae that could lead to secondary elevated intraocular pressure, all cataract surgeries were combined with iridectomy in the superior temporal or superior nasal quadrant. Some children also underwent anterior vitrectomy and posterior capsulotomy. Following cataract surgery, a 700 µg dexamethasone implant was implanted intravitreally at 3.5 mm from the corneal edge.

### Data analysis

SPSS version 22 statistical software package was used for data analysis. Descriptive analysis was used for qualitative data evaluation. The continuous variables were described as means and standard errors of the mean (SEM) or median and interquartile range (IQR). Visual (histograms, probability plots) and analytical (Kolmogorov-Smirnov/Shapiro-Wilk tests) methods were employed to investigate variables for normality distribution. Multiple groups significance analysis for multiple comparisons was performed using analysis of variance (ANOVA) test. The two groups were compared using the independent-sample t-test and Mann–Whitney statistical test. In all statistical analyses, *p* ≤ 0.05 was considered statistically significant.

## Results

After meeting the inclusion and exclusion criteria, a total of 36 eyes from 28 patients underwent pediatric cataract phacoemulsification combined with intraocular lens implantation and simultaneous injection of dexamethasone implantation in the study. Eight patient underwent bilateral surgery. The mean age of children diagnosed with uveitis was 8.67 ± 3.09 years. The mean age of children undergoing cataract surgery was 11.07 ± 4.01 years. Among the included children, there were 11 males and 17 females. Among them, 7 children were diagnosed with JIA-associated uveitis, while the rest had idiopathic pediatric uveitis.

Prior to undergoing cataract surgery, 15 children (53.6%) received oral prednisone therapy (range 5–15 mg/day). All children received methotrexate therapy (range 0.125–0.5 g/day). Additionally, 20 children received biologic therapy with subcutaneous injections of adalimumab (40 mg every 2 weeks). Demographic, clinical, and treatment details are listed in Tables 1 and 2.

**Table 1:** Baseline characteristics for eyes with pediatric uveitis before cataract surgery

	Baseline Characteristics (*N* = 28)
Age at uveitis diagnosis, mean (SD), years	8.67 (3.09)
Age at cataract diagnosis, mean (SD), years	11.07 (4.01)
Time to cataract diagnosis, median (IQR), months	16.00 (10.00–40.00)
Sex (Male/Female)	11/17
Uveitis diagnosis, n(%)	
JIA	7 (25%)
Idiopathic	21 (75%)
Baseline BCVA, median (IQR), logMAR	1.00 (0.40–1.50)
Baseline IOP, mean (SD), mmHg	12.85 (4.61)
Other ocular feature, n(%)	
Posterior synechiae	28 (100%)
Bank-shaped keratopathy	11 (39.3%)
Glaucoma	1 (3.6%)

JIA: juvenile idiopathic arthritis -associated uveitis; IOP: intraocular pressure; BCVA: best-corrected visual acuity

**Fig. 1:**
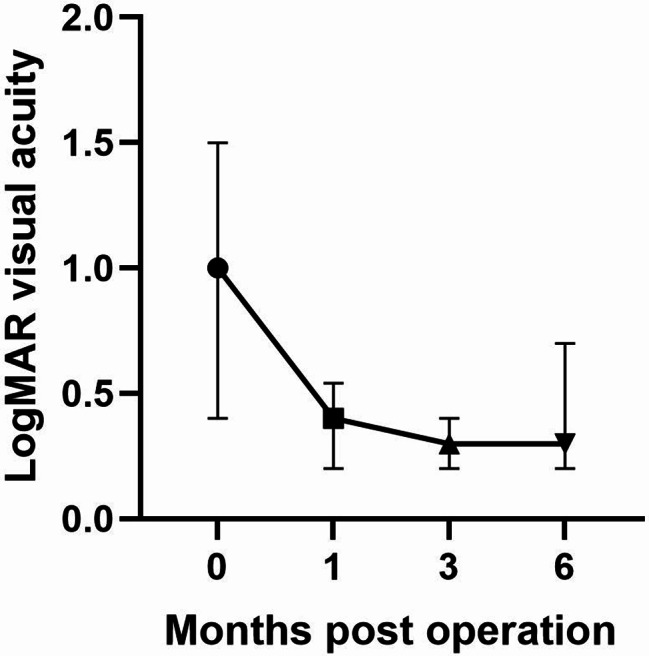
The trend profile of visual acuity on various follow-up visits before and after cataract surgery

The mean preoperative BCVA was 1.00 (0.40–1.50) LogMAR. BCVA significantly improved to 0.40 (0.20–0.54) LogMAR at 1 month postoperatively (*P* = 0.006), further improving to 0.30 (0.20–0.40) LogMAR at 3 months postoperatively (*P* = 0.001). BCVA remained stable at 0.30 (0.20–0.70) LogMAR at 6 months postoperatively (*P* = 0.005). During the 6-month follow-up, there was no decrease in BCVA compared to preoperative values in any eye. The results of BCVA are presented in Table 2; Fig. 1.

**Table 2:** Clinical and treatment details of children before and after cataract surgery with intravitreal dexamethasone implant

	Pre-operation	1 month	3 months	6 months
BCVA, median (IQR), logMAR	1.00 (0.40–1.50)	0.40 (0.20–0.54)^*^	0.30 (0.20–0.40)^*^	0.30 (0.20–0.70)^*^
IOP, mean (SD), mmHg	12.85 (4.61)	13.23 (3.75)	11.93 (3.59)	12.87 (3.85)
Oral glucocorticoids, n (%)	15 (53.6%)	15 (53.6%)	11 (39.3%)	13 (46.4%)
Mycophenolate mofetil, n (%)	28 (100%)	28 (100%)	28 (100%)	28 (100%)
Adalinumab, 40 mg 2 weekly, n (%)	20 (71.4%)	22 (78.6%)	26 (92.9%)	28 (100%)
Dosage of oral glucocorticoids, median (IQR), mg/d	10.00 (5.00-11.88)	10.00 (5.00-13.13)	5.00 (5.00-5.63)	5.00 (5.00–10.00)
Dosage of mycophenolate mofetil, median (IQR), n (%)	0.25 (0.25–0.38)	0.25 (0.25–0.38)	0.25 (0.25–0.25)	0.25 (0.25–0.25)

IOP: intraocular pressure; BCVA: best-corrected visual acuity

^*^P < 0.05

**Fig. 2:**
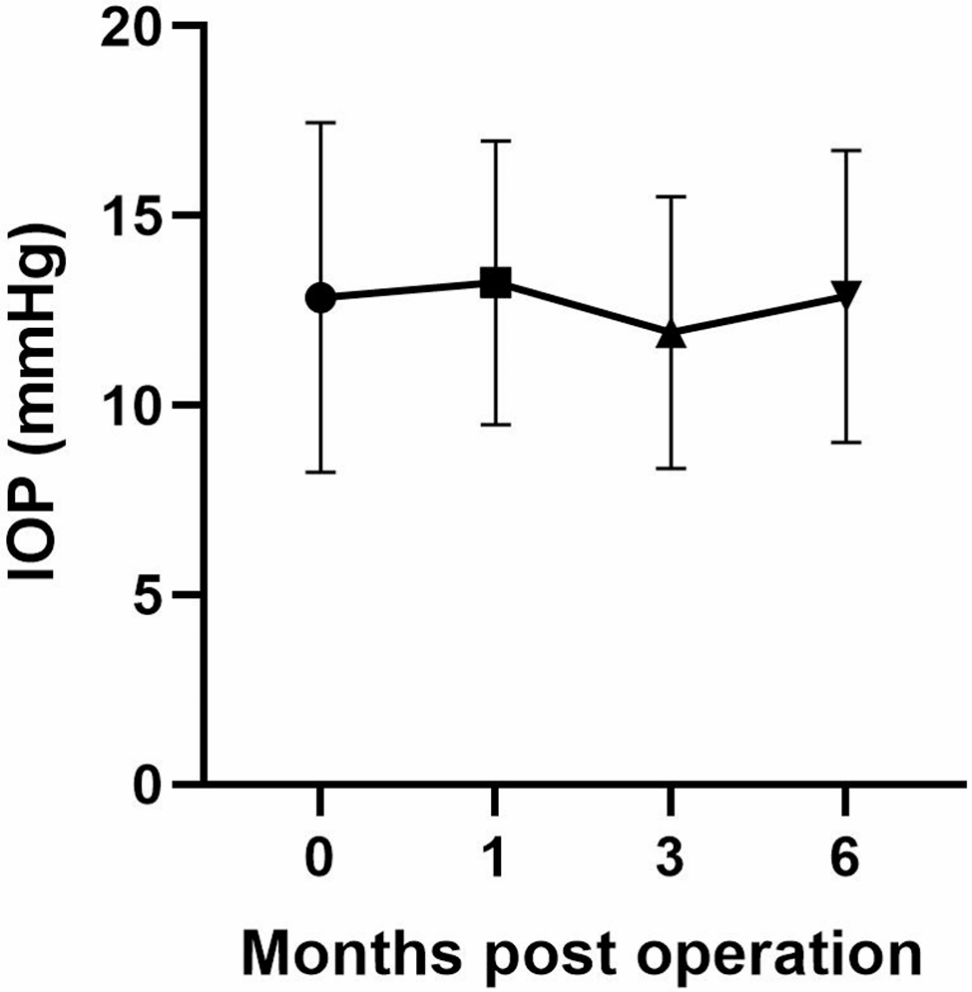
The trend profile of IOP on various follow-up visits before and after cataract surgery

The mean preoperative IOP was 12.85 ± 4.61 mm Hg. There was no significant change in IOP during the 6-month postoperative follow-up compared to preoperative values. The mean IOP was 13.23 ± 3.75 mmHg at 1 month postoperatively (*P* = 0.987), 11.93 ± 3.59 mmHg at 3 months postoperatively (*P* = 0.862), and 12.87 ± 3.85 mm Hg at 6 months postoperatively (*P* > 0.99). Changes in IOP are shown in Table 2; Fig. 2. None of the children experienced elevated IOP or developed glaucoma during the follow-up period. No ocular or systemic side effects were observed in any child during the treatment process.

During follow-up, we found that 8 children experienced recurrence of ocular inflammation during the follow-up period of three to six months after surgery. These children presented with varying degrees of visual acuity decline and inflammation cells in the anterior chamber and vitreous cavity. Depending on the severity of the inflammatory response, treatment options included oral prednisone therapy, periocular steroid injections, or topical steroid eye drops. Specific treatment regimens and ocular conditions are detailed in Table 3 (end of the manuscript file). No cases of macular edema were observed in any eye during the 6-month postoperative follow-up.

**Table 3:** Clinical and treatment details of children experienced recurrence of ocular inflammation

Patient	Eye	Sex	Age at uveitis diagnosis (years)	Age at surgery (years)	Preop BCVA/IOP	Inflammation control	1-month BCVA/ IOP	Inflammation control	3-month BCVA/IOP	Inflammation control	6-month BCVA/ IOP	Inflammation control
1	OD	M	14	15	1.3/19.8	Prednisone 12.5 mg/d + MMF 0.5 g/d + ADA 40 mg/2 weeks	0.54/8.4	Prednisone 12.5 mg/d + MMF 0.5 g/d + ADA 40 mg/2 weeks	0.4/8.4	Prednisone 10 mg/d + MMF 0.5 g/d + ADA 40 mg/2 weeks	1.3/5.7	Prednisone 15 mg/d + MMF 0.5 g/d + ADA 40 mg/2 weeks + periocular corticosteroid injections + corticosteroid eye drops
2	OD	F	4	5	3/14.5	Prednisone 5 mg/d + MMF 0.25 g/d	0.54/14.1	Prednisone 5 mg/d + MMF 0.25 g/d	0.2/10.6	MMF 0.25 g/d	0.7/8.4	MMF 0.5 g/d + ADA 40 mg/2 weeks + corticosteroid eye drops
3	OS	F	8	15	1.1/10.6	MMF 0.25 g/d + ADA 40 mg/2 weeks	0.5/10.5	Prednisone 15 mg/d + MMF 0.25 g/d + ADA 40 mg/2 weeks	0.2/8.1	Prednisone 5 mg/d + MMF 0.25 g/d + ADA 40 mg/2 weeks	0.54/13.2	Prednisone 5 mg/d + MMF 0.25 g/d + ADA 40 mg/2 weeks + corticosteroid eye drops
4	OS	F	11	12	1/21.9	MMF 0.25 g/d + ADA 40 mg/2 weeks	0.54/7.1	MMF 0.25 g/d + ADA 40 mg/2 weeks	0.4/6.8	MMF 0.25 g/d + ADA 40 mg/2 weeks	0.54/7.3	MMF 0.25 g/d + ADA 40 mg/2 weeks + corticosteroid eye drops
5	OD	F	5	8	0.7/8.6	Prednisone 5 mg/d + MMF 0.125 g/d + ADA 40 mg/2 weeks	0.4/15.1	Prednisone 5 mg/d + MMF 0.125 g/d + ADA 40 mg/2 weeks	0.2/14.4	MMF 0.125 g/d + ADA 40 mg/2 weeks	0.7/13.2	Prednisone 5 mg/d + MMF 0.125 g/d + ADA 40 mg/2 weeks + corticosteroid eye drops
6	OS	M	10	11	1.6/7	Prednisone 12.5 mg/d + MMF 0.25 g/d + ADA 40 mg/2 weeks	1.3/18.4	Prednisone 12.5 mg/d + MMF 0.25 g/d + ADA 40 mg/2 weeks	1.18/16	Prednisone 5 mg/d + MMF 0.25 g/d + ADA 40 mg/2 weeks	1.3/15	Prednisone 10 mg/d + MMF 0.5 g/d + ADA 40 mg/2 weeks + corticosteroid eye drops
7	OS	M	8	13	0.4/13.1	Prednisone 5 mg/d + MMF 0.25 g/d + ADA 40 mg/2 weeks	0.2/15	Prednisone 5 mg/d + MMF 0.25 g/d + ADA 40 mg/2 weeks	0.1/10.5	MMF 0.25 g/d + ADA 40 mg/2 weeks	0.2/13.1	MMF 0.25 g/d + ADA 40 mg/2 weeks + corticosteroid eye drops
8	OS	M	6	7	1.0/10.8	MMF 0.25 g/d + ADA 40 mg/2 weeks	0.1/11.6	MMF 0.25 g/d + ADA 40 mg/2 weeks	0.1/16.9	MMF 0.25 g/d + ADA 40 mg/2 weeks + corticosteroid eye drops	0.54/17.0	Prednisone 10 mg/d + MMF 0.25 g/d + ADA 40 mg/2 weeks + periocular corticosteroid injections

## Discussion

Research indicates that the estimated interval time from diagnosis of uveitis to cataract extraction in children with uveitis is 3.4 to 4.5 years, making cataracts the most common ocular complication in children with uveitis [12–14]. The most critical consideration in pediatric uveitis is amblyopia as a sequelae of the disease, particularly for younger patients in the developmental stages of vision (under 8 years old), necessitating proactive intervention by ophthalmologists. The most significant prognostic factor affecting outcomes of pediatric cataract surgery is postoperative inflammation control. This study found that intraoperative intravitreal dexamethasone implantation is a safe and effective method for preventing and reducing postoperative inflammation in children with uveitis undergoing cataract surgery. All patients maintained systemic immunomodulatory therapy, with no need for immediate pre- or postoperative oral corticosteroid supplementation. However, some children experienced recurrence of ocular inflammation after 3 months.

There is limited research on the use of intravitreal steroid implants in controlling post-cataract surgery inflammation in uveitis. Gupta [15] and colleagues observed the safety and efficacy of intravitreal dexamethasone implantation during cataract surgery in patients with uveitis. In comparison to the standard care group, all patients in the dexamethasone implant group had visual acuity equal to or better than 6/12, while only 70% of patients in the standard care group achieved this level. Additionally, no cases of cystoid macular edema (CME) occurred in the dexamethasone implant group, whereas the incidence of CME in the standard treatment group was 37.5%. In another comparison between the dexamethasone implantation group and the oral steroid group, there was no statistically significant difference in BCVA, IOP, and CMT between the two groups postoperatively. Neither group exhibited significant elevation in IOP [16]. This suggests that a single intravitreal dexamethasone implantation may be an effective alternative to postoperative oral steroids. Jinagal [9] evaluated the safety and efficacy of intravitreal dexamethasone implantation in JIA-associated uveitis in a retrospective case series of pediatric uveitis. Significant improvement in BCVA was observed at 1, 3, and 6 months postoperatively in 8 eyes of 6 patients. Mean IOP did not differ significantly at various follow-up intervals. No cases of uveitis or worsening of macular edema were reported.

In our study, all children showed significant improvement in BCVA at the 6-month follow-up compared to preoperative values. At 3 months, the overall BCVA was the best, with a median (IQR) of 0.30 LogMAR (0.20 LogMAR − 0.40 LogMAR). Some children experienced recurrence of ocular inflammation at 6 months, resulting in visual acuity decline. Previous studies did not observe recurrence of inflammation using dexamethasone implantation to control post-cataract surgery inflammation in pediatric uveitis. However, our research has found that even with dexamethasone implantation, some children with pediatric uveitis may experience recurrence of ocular inflammation. In a retrospective study involving 14 eyes of 11 children with uveitis, Taylor [17] and colleagues assessed the efficacy of intravitreal dexamethasone implant in controlling inflammation in pediatric uveitis. Within 1 month post-injection, inflammation was controlled and/or macular edema resolved in 13 eyes (93%), but 4 eyes (31%) experienced recurrence within 6 months, with a median time to recurrence of 4 months (range 3–5 months). Sella and Bratton [18, 19] also found that most children with uveitis experienced improvement in vision and inflammation control within 1–3 months post-injection, but approximately half experienced recurrence of inflammation 4–6 months post-injection, necessitating re-implantation of dexamethasone implantation or increased use of local steroids. We believe this may be related to factors such as the metabolism time of dexamethasone in the eye, the severity of pediatric uveitis, and systemic medication usage. Studies have shown that the improvement in vision and resolution of macular edema achieved with intravitreal injections of dexamethasone implantation may dissipate after 6 months in pediatric uveitis [20]. While this study found that dexamethasone implantation also effectively controls inflammation in pediatric uveitis after cataract surgery, we still recommend that ophthalmologists follow up regularly after surgery, especially after 3 months, to prevent recurrence of inflammation.

In terms of controlling postoperative complications, dexamethasone implantation also showed excellent results. No cases of CME or steroid-induced elevated IOP or glaucoma were observed in any child. The most significant safety concern with any ocular corticosteroid treatment is elevated IOP. Particularly in children, corticosteroid-induced ocular hypertension tends to occur more frequently and rapidly. In this study, none of the children required IOP-lowering treatment during the 6-month follow-up. Our study suggests that the use of intravitreal dexamethasone implantation in pediatric uveitis cataract surgery is safe. This may also be attributed to the peripheral iridectomy performed in every child. The reported incidence of glaucoma or elevated IOP after pediatric uveitis cataract surgery varies widely, from 20–80%.[21–23] Sanna and colleagues [24] reported 20 cases of JIA-associated uveitis cataracts removed and intraocular lenses implanted. During a 5-10-year follow-up, iris posterior synechiae or pupillary membranes recurred in 10 eyes (38%), and glaucoma or elevated IOP occurred in 21 eyes (81%). We recommend performing peripheral iridectomy during cataract surgery to reduce the likelihood of future glaucoma, but our experience requires long-term follow-up to confirm.

The limitations of this study include its retrospective design and small sample size, limiting statistical analysis and necessitating larger studies to validate these findings. Another limitation is the lack of a comparison group, i.e., children with uveitis undergoing phacoemulsification and intraocular lens implantation under standard care (oral corticosteroids). Prospective studies are needed to overcome this limitation.

## Conclusion

In conclusion, intravitreal dexamethasone implantation combined with phacoemulsification and intraocular lens implantation appears to be a safe and feasible option for children with uveitis. This approach effectively controls postoperative inflammation while avoiding the need for increased systemic corticosteroids during the perioperative period. However, some children may experience recurrence of inflammation after 3 months following cataract surgery, necessitating close follow-up and monitoring by clinicians for postoperative complications and recurrence of ocular inflammation. Future prospective studies with larger sample sizes may help further understand the differences in the occurrence rates of ocular inflammation control and complications post-cataract surgery in pediatric uveitis between intravitreal dexamethasone implantation and systemic corticosteroids.

## Data Availability

No datasets were generated or analysed during the current study.

## Abbreviations

JIA: juvenile idiopathic arthritis
BCVA: best-corrected visual acuity
ADA: Adalimumab
SUN: Standardization of Uveitis Nomenclature
IOP: intraocular pressure
EDI-OCT: enhanced depth imaging optical coherence tomography
FFA: fundus fluorescein angiography
ICGA: indocyanine green angiography
PPD: Purified Protein Derivative
QFT: QuantiFERON-TB Gold test
CME: cystoid macular edema
